# Chromosome-level assembly and phylophenetic insights of *Cladosporium oxysporum* A3.I.1, a fungus with the ability to degrade polyurethane polymers

**DOI:** 10.1093/g3journal/jkag025

**Published:** 2026-02-26

**Authors:** Ayixon Sánchez-Reyes, Itzayana Chavarría-Quintanilla, Martín Vargas-Suárez, Miguel A Cevallos, Itzel Gaytán, Herminia Loza-Tavera

**Affiliations:** Investigador por México, SECIHTI. Instituto de Biotecnología, Universidad Nacional Autónoma de México, 62210 Cuernavaca, Morelos, México; Departamento de Bioquímica, Facultad de Química, Universidad Nacional Autónoma de México, 04510 Coyoacán, Ciudad de México, México; Departamento de Bioquímica, Facultad de Química, Universidad Nacional Autónoma de México, 04510 Coyoacán, Ciudad de México, México; Programa de Genómica Evolutiva, Centro de Ciencias Genómicas, Universidad Nacional Autónoma de México, 62210 Cuernavaca, Morelos, México; Departamento de Procesos y Tecnología, División de Ciencias Naturales e Ingeniería, Universidad Autónoma Metropolitana, Unidad Cuajimalpa, 05348 Cuajimalpa, Ciudad de México, México; Departamento de Bioquímica, Facultad de Química, Universidad Nacional Autónoma de México, 04510 Coyoacán, Ciudad de México, México

**Keywords:** fungal genomics, molecular phylogenetics, *Cladosporium oxysporum* A3.I.1, plastic biodegradation, speciation

## Abstract

Notwithstanding the ecological significance of the *Cladosporium* genus, which includes several cosmopolitan fungi reported as saprobes, plant pathogens, and causes of allergic and cutaneous diseases in humans, only 41 genomes have been reported. Here, we report the genome of a polyurethane (PU)-degrading fungus initially identified as *Cladosporium tenuissimum* A3.I.1. PU is a plastic material widely used as mattress foams, building insulation, protective sealants, and fabrics, that is highly resistant to degradation and barely recycled, hence its waste remains in the environment for hundreds of years. After sequencing its genome using three different techniques—Illumina, PacBio SMRT RSII, and Hi-C—and performing different bioinformatic analyses, the A3.I.1 species placement was reassessed. This analysis provides the first high-quality chromosome-level genome assembly, with 33,552,215 bp, a genome coverage of 58.7X, a contig N50 of 1,881,040 bp, and a GC content of 53%. Assembly scaffolding with Proximo Hi-C revealed 19 scaffolds, corresponding to the total number of haploid chromosomes. The phylophenetic analysis supports its re-classification as *Cladosporium oxysporum* strain A3.I.1, refining its taxonomic placement. When comparing A3.I.1 against the reference genome of *C. oxysporum* strain AAAS_A1, 693,670 single-nucleotide variants, 18,623 insertions, and 18,687 deletions were uncovered in the A3.I.1 genome. Gene prediction using FunGAP identified 13,115 protein-coding genes, 121 tRNA genes, and 69 rRNA genes. This genomic resource provides valuable insights into environmental resilience research and supports the emerging role of *C. oxysporum* A3.I.1 as a PU degrader. It also opens new venues for the development of sustainable biorecycling processes.

## Introduction

The *Cladosporium* genus, a cosmopolitan hyphomycete, includes species widely recognized for their roles in environmental ecosystems and as opportunistic pathogens. It has a long history of study, primarily due to its impact on agriculture and human health, and some species have been recognized as allergens (Dugan et al. 2004; Heuchert et al. 2005). However, despite its ecological relevance, genomic resources for the *Cladosporium* genus have remained limited, with only 41 genomes reported in the NCBI database (O'Leary et al. 2024), from >230 species already described (Iturrieta-González et al. 2021), hindering deeper insights into its biology, functional potential, and evolution.

The motivation for compiling the dataset presented in this work stemmed from the need to understand better the genomic structure and assembly, as well as the speciation landscape, of an environmental fungal isolate initially collected for its ability to colonize polyurethane (PU) foam wall coverings and subsequently studied for its capability to degrade polyester PU coatings and polyether PU foams (Álvarez-Barragán et al. 2016). Initially, it was identified as *Cladosporium tenuissimum* strain A3.I.1 based on the ITS1-D2 rRNA sequence and partial sequencing of the actin and translation elongation factor 1 genes (Álvarez-Barragán et al. 2016). In this work, based on genome sequencing data, this isolate has been re-evaluated in the context of the genomic representatives of the *Cladosporium* genus, to clarify its taxonomic placement, based on the phylophenetic species concept, which combines phylogenomic reconstruction with species delimitation using the Poisson Tree Processes model (Rosselló-Mora and Amann 2001; Zhang et al. 2013; Ide-Pérez et al. 2024).

The theoretical background of this work is rooted in comparative genomics and phylogenetics, which seek to understand species relationships at the genomic level. Methodologically, advancements in sequencing technologies, including long-read sequencing and chromosomal assembly techniques, have enabled the generation of high-resolution genomic datasets. These developments have opened new avenues for studying genome structure, gene content, and the evolutionary relationships of fungal species. In this context, the decision to assemble a chromosome-level genome for the strain A3.I.1 was made to fill a critical gap in the current genomic resources. The main goals of this work were to (i) generate a high-quality chromosome-level genome assembly; (ii) contextualize this genome within the current taxogenomic framework of the *Cladosporium* genus; and (iii) provide foundational data for future research in fungal biology, environmental adaptability, and evolution.

This new resource provides a comprehensive reference for comparative genomic studies, enabling accurate gene annotation, evolutionary analyses, detection of structural variation, and investigation of traits related to environmental adaptation and biodegradation of PU. This assembly enables research in fungal ecology, phylogenetics, and biotechnology, and provides the genetic basis for the development of bioremediation processes in PU waste management.

## Methods

### Fungal cultivation and DNA extraction

Origin and sampling of the strain A3.I.1 were previously described (Álvarez-Barragán et al. 2016). Fungal cultures were established by inoculating 50 μL of conidial suspension into 25 mL of potato dextrose broth, and incubating at 30 °C for 7 d. Mycelium was collected by filtration, washed three times with 50 mM phosphate buffer (pH 6.5), and excess moisture was removed using filter paper. The harvested mycelium was then frozen and ground under liquid nitrogen. Genomic DNA was extracted using the E.Z.N.A. HP Fungal DNA Kit (Omega Bio-Tek) following the manufacturer's protocol.

### Genome sequencing

Three complementary sequencing strategies were employed to maximize assembly contiguity and completeness. Short-read shotgun sequencing was performed in the Illumina HiSeq 2500 Rapid 100PE platform, using 6.7 µg of gDNA, generating ∼100X genome coverage (Macrogen, USA). Illumina reads were initially pre-filtered by the sequencing provider. Quality assessment was performed using FastQC (v0.11.7), which confirmed high base-call accuracy and no sequences flagged as poor quality. The dataset comprised 69,627,836 reads encoded in Sanger/Illumina 1.9 format. Overall, 96.98% of bases exceeded Q20 and 94.91% exceeded Q30 thresholds. For downstream analysis, only reads with base quality scores of Q30 or higher were retained.

Long-read sequencing was performed on the PacBio SMRT RSII system at the Yale Center for Genome Analysis (Yale University, USA). The quality of the starting genomic DNA (gDNA) was evaluated by Nanodrop and Qubit measurements. The A_260_/A_280_ and A_260_/A_230_ ratios were both >1.8. The size of the dDNA was evaluated by Pippin Pulse Electrophoresis system (Sage Science, USA), finding that a significant portion of the gDNA migrated above 30 to 40 kb. In a Covaris G-Tube device, the input gDNA (10 μg) was sheared, it was passed through AmPure beads for size selection (10 to 20 kb) and recovered in PacBio Elution Buffer. The concentration and size of the sheared DNA were checked by Qubit dsDNA HS Assay kit and Pippin Pulse gel electrophoresis. After damage repair and ends repaired, fragmented DNA was ligated with PacBio adaptors to make the SMRTbell libraries. Exonuclease digestion was performed to remove partial ligation products. SMRTbell libraries were then annealed to sequencing primers and then bound to polymerase before loading on the PacBio RSII sequencer. Sequencing was performed for 6 h movie time, across three SMRT cells. HDF5, fasta and fastq files were generated during the sequencing and transferred to HPC data storage server. Raw reads were adapter-trimmed and quality-filtered and used for de novo genome assembly using RS_HGAP_Assembly.3 in the SMRT Portal Analysis version 2.3.0. The sequencing data yielded 256,305 reads, totaling 3.46 Gb, achieving ∼100X genome coverage.

For the Hi-C analysis, 200 mg of mycelium was harvested and incubated in 1% formaldehyde for 20 min. After that, 0.1 g of glycine was added and mixed until homogenized, incubating for 15 min with mixing by inversion every three minutes. Then, the mycelia were washed twice with distilled water by centrifuging 1,000*×g* per 1 min. The tissue was sent to Phase Genomics, Inc. (Seattle, WA, USA). For details about the sequencing techniques, the provider's protocol (https://info.phasegenomics.com/hubfs/Protocol_Fungal_v4.5_202404.pdf) can be consulted. The results were analyzed with the Proximo Genome Scaffolding, a Phase Genomics (https://phasegenomics.com) proprietary software.

### Genome assembly and phylogenetic analysis

A two-step assembly approach was implemented. First, a hybrid assembly combining Illumina and PacBio reads was performed using SPAdes version 3.14.1 (https://github.com/ablab/spades/tree/v3.14.1), executed via the Linux/Ubuntu-compatible command-line interface (Bankevich et al. 2012), with the --pacbio flag and -1/-2 options used to specify read type and paired-end inputs, respectively. All other parameters were set to their default values. Subsequently, Hi-C reads were used to order and orient contigs, achieving chromosome-scale scaffolding with the proprietary software Proximo: Genome Scaffolding (Phase Genomics. Bioinformatics Phase Genomics Technology. https://phasegenomics.com/technology/bioinformatics/ (accessed July 05, 2025)). Assembly completeness and quality were assessed using BUSCO version 5.8.2 (https://busco.ezlab.org/) and QUAST version 5.3.0 (Gurevich et al. 2013; Manni et al. 2021) in the Ascomycota_odb12 datasets with default parameters. Phylogenetic reconstruction was performed using JolyTree version 2.1.211019ac, a distance-based method for inferring phylogenies without prior multiple sequence alignments (Criscuolo 2019) with default options. The k-mer size was automatically estimated as –k = 28 based on the largest genome (*Cladosporium* sp. IMV 00045, GCA_001931905.2). To further evaluate species boundaries, a Bayesian Poisson Tree Processes (bPTP) analysis was applied to the phylogenetic tree (JolyTree), leveraging likelihood-based partitions to infer putative species-level delimitations (Zhang et al. 2013). The analysis employed Markov Chain Monte Carlo (MCMC) sampling with a fixed seed value (SEED = 1234) and 1,000,000 iterations to ensure convergence; all other parameters were set to default values. Tree display and annotation were performed with Interactive Tree Of Life (iTOL) version 5.7 (Letunic and Bork 2021).

To assess the congruence of the genome-based reference tree (JolyTree), a multi-gene phylogenetic tree using the Up-to-date Fungal Core Genes dataset (UFCG version 1.0.6 for Unix systems) (Kim et al. 2023) was constructed. To this end, we applied three complementary metrics. The Gene concordance factor (gCF) was calculated using the multicore version 2.4.0 of IQ-TREE for Linux x86_64 to estimate the proportion of individual gene trees supporting the reference topology (Nguyen et al. 2015). Tree Distance was used to quantify normalized topological divergence between the two trees. Additionally, NyeSimilarity was computed to evaluate shared structural features. These metrics were computed in R using the TreeDist package (version 2.11.1) (Smith 2020).

### Gene prediction and annotation

Protein-coding genes, including introns and exons, were predicted using FunGAP (Fungal Genome Annotation Pipeline version 1.1.1) (https://github.com/CompSynBioLab-KoreaUniv/FunGAP) (Min et al. 2017) with default parameters. Functional annotation was performed by assigning orthologous genes using the KEGG Automatic Annotation Server (KAAS). For pathway reconstruction and functional classification, the KEGG Mapper (Moriya et al. 2007; Kanehisa and Sato 2020) was employed. Hexamer frequency estimation was performed using FOCUS version 1.6 (Silva et al. 2014) with default parameters to assess k-mer composition and genomic sequence signatures. k-mer spectra, analysis of repetitive DNA, and heterozygosity level estimates were obtained using GenomeScope2 (Ranallo-Benavidez et al. 2020) under default options. Telomeric repeats were identified using tidk version 0.2.65 (Brown et al. 2025) with default settings to locate and characterize putative telomeric regions in the assembled genome. The mitochondrial genome of strain A3.I.1 was annotated using MITOS2 version 1.1.3 (Bernt et al. 2013), which was implemented in the Proksee web server for structural feature mapping (Grant et al. 2023). Structural variations between strain A3.I.1 and the reference sequence (GCA_035771495) were identified using GSAlign version 1.0.22 with default settings (Lin and Hsu 2020). All computational analyses were performed in a Debian GNU/Linux 12 (bookworm) environment via terminal, unless otherwise stated.

## Results and discussion

### Genome assembly

**Fig. 1.**
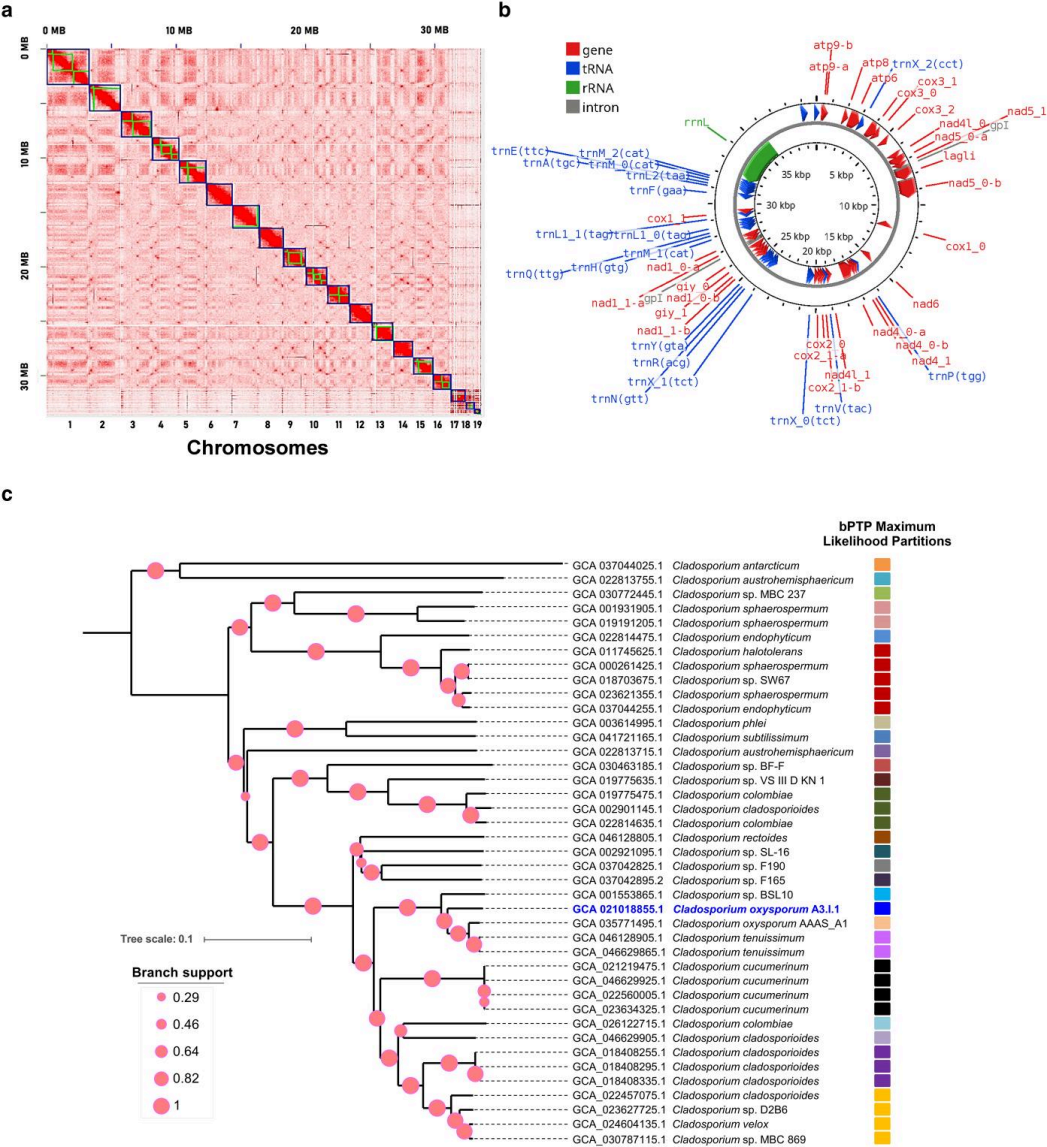
Integrated genomic overview of strain A3.I.1: chromosomes, mitogenome, and phylogeny. a) Chromosome Hi-C contact map of *Cladosporium oxysporum* A3.I.1 genome after scaffolding, showing 19 quasi-chromosomes ordered by size and depicted in blue, with scale bars in megabases (MB). b) Circular map of the assembled and annotated mitochondrial genome. The mitogenome was annotated using MITOS2 and visualized with Proksee. Annotated features include protein-coding genes, transfer RNAs, ribosomal RNAs, and introns. c) Phylogenomic tree based on whole-genome distances. Branch supports are depicted as circles at scale. Tree scale represents changes per site. The column with colored squares represents partitions identified by the species delimitation analysis using the Bayesian Poisson Tree Processes (bPTP) model. Putative species boundaries are indicated along branches using different colors; the same color corresponds to the same species. *C. oxysporum* strain A3.I.1and the reference *C. oxysporum* strain AAAS_A1 (GCA_035771495.1) form separate partitions in the bPTP analysis but are still considered conspecifics, representing intra-specific lineages.

The genome of the strain A3.I.1 was assembled using Illumina HiSeq reads, PacBio RSII long reads, and high-throughput chromosome conformation capture (Hi-C) data. A high-quality chromosome-level genome of 33,552,215 bp was generated (NCBI genome assembly ASM2101885v1). The assembly achieved a sequencing coverage of 58.7X, with a GC content of 53%, and exhibited high assembly quality, as indicated by its N50 value of 1,881,040 bp (Table 1). The assembly scaffolding using the Proximo Hi-C platform organized the genome into 19 scaffolds, which corresponded to the total number of haploid chromosomes, as shown in the Hi-C contact data matrix (Fig. 1a). Assembly integrity, completeness, and redundancy analyses revealed that 98.73% of BUSCOs were complete, comprising 98.45% single-copy genes, and 0.28% duplicates; additionally, 0.25% were fragmented genes, and 1.02% were missing BUSCOs. In addition, we identified five distinct telomeric sequences types within the assembly (https://doi.org/10.6084/m9.figshare.29755565.v5) (Sánchez-Reyes 2025). The telomeres and their positions in the genome sequence were not identified. After the Phase Genomics Assembly, we obtained 19 scaffolds, corresponding to the chromosomes and several contigs representing 0.45% of the total input assembly. Those contigs could not be incorporated into the chromosome scaffolds because they were too short or too repetitive to have a clear signal in the Hi-C data. Since repetitive sequences are characteristic of telomeres, we deduce that these contigs correspond to telomeres, but the assembly program could not associate them to specific chromosomes.

**Table 1.** Summary statistics for the *Cladosporium oxysporum* A3.I.1 assembly and annotation.

Organism name	*Cladosporium oxysporum*
Infraspecific name	Strain: A3.I.1
BioSample	SAMN16055832
BioProject	PRJNA661343
Genome representation	full
Assembly accession	GCA_021018855.1 (latest)
WGS Project	JAIQXY01
Assembly method	SPAdes v. 3.14.1 RS_HGAP_Assembly.3 Proximo Genome Scaffolding v. 39a526e
Genome coverage	58.7X
Sequencing technology	Illumina HiSeq; PacBio SMRT RSII; Hi-C Seq
Total sequence length	33,552,215 bp
Mitochondrial genome length	31,589 bp
Number of contigs	19 nuclear 1 mitochondrial
Contig N50	1,881,040 bp
Contig L50	8
N's	6909
G + C content (%)	53
Annotation predictions	
Protein-coding genes	13,115
tRNAs	121
rRNAs	69
BUSCO completeness (%)	98.73
KEGG Orthology (KO)	5,518

The GenomeScope2 analysis revealed a haploid-like genome structure (Supplementary Fig. 1). Additionally, the analysis estimated that the optimal haploid genome length was 38,255,053 bp, comprising a unique length of 31,971,257 bp (83.6%) and 6,283,796 bp of repetitive regions, achieving a model fit accuracy of 96.49%. These results suggest a well-resolved and reliable genomic reconstruction (Supplementary Table 1). A mitochondrial genome of 31,589 bp was also assembled (Table 1, Fig. 1b).

### Phylogeny and genome annotation

The phylogenetic reconstruction generated with JolyTree and refined in iTOL showed that the A3.I.1 strain, previously classified based on its ITS sequence as *C. tenuissimum*, clustered instead within the clade of *Cladosporium oxysporum* reference strain AAAS_A1 (Accession number: GCA_035771495.1). In the nineteenth century, *C. oxysporum* Berk. & M.A. Curtis was first described growing on dead leaves of *Passiflora* sp. in Cuba (Berkeley and Curtis 1868). It is a common saprobe with a broad ecological distribution, including soil and plant surfaces (Schubert and Braun 2005; Bensch et al. 2012; Borman and Johnson 2023 ). Only the genome assembly of the *C. oxysporum* AAAS_A1 strain, a plant pathogen, has been reported; it is highly fragmented (52 contigs), limiting the resolution required for detailed comparative genomic, taxogenomic, and evolutionary studies. Consequently, the genomic landscape of *C. oxysporum* remains unexplored, hindering broader insights into its functional genomics, adaptation mechanisms, and evolutionary relationships within the genus. The *C. oxysporum* A3.I.1 genome assembly had superior integrity compared to that of strain AAAS_A1.

As inferred from the intrachromosomal contact probability maps generated by Hi-C sequencing analysis, which provide reliable information, the A3.I.1 strain presented a haploid chromosome number of 19. Although 97.11% of the shared hexamer frequency between A3.I.1 and AAAS_A1 strains indicates a remarkable similarity in the structural organization of their coding regions, subtle differences may still reflect lineage-specific adaptations. A comparative analysis using A3.I.1 as a query, against the reference genome of *C. oxysporum* strain AAAS_A1, revealed 693,670 single-nucleotide variants (SNVs), 18,623 insertions, and 18,687 deletions in the A3.I.1 genome (A3i1q_vs_AAASr.vcf in: https://doi.org/10.6084/m9.figshare.29755565.v5) (Sánchez-Reyes 2025). These structural variations highlight a genetic divergence from the reference strain, suggesting potential evolutionary pressures, niche adaptation, or functional specialization within the *C. oxysporum* clade.

Moreover, the species delimitation analysis (bPTP likelihood partitions) suggests that A3.I.1 may represent a divergent line within *C. oxysporum*. While this result points to potential cryptic variation, we acknowledge that robust species delimitation would require additional analyses (*e.g*. multilocus data and population-level genetic diversity) to support any formal taxonomic reassignment. At present, the most parsimonious and genomically coherent hypothesis is to include the A3.I.1 strain into *C. oxysporum*, consistent with current phylogenomic evidence (Fig. 1c). This phylophenetic analysis resolves the previous misidentification, and it emphasizes the power of taxogenomics in clarifying species relationships beyond traditional methods based on the ITS. It also reveals potential misclassifications in the genus, where isolates assigned to *C. cladosporioides* and *C. colombiae* appear in multiple clades, suggesting that some strains may require reassignment or that these taxa comprise polyphyletic lineages. Overall, the tree highlights the need for taxonomic reevaluation within the *Cladosporium* genus, supported by genome-wide data and modern species delimitation approaches (Fig. 1c).

To further validate the corrected species identification of strain A3.I.1 as *C. oxysporum*, we reconstructed a UFCG-based phylogeny and performed a concordance analysis using IQ-TREE. As expected, the results demonstrated strong topological agreement with our JolyTree-based species tree, as indicated by a gCF of 84.21% and a gene discordance factor due to polyphyly of <5.26%. Additionally, the Robinson–Foulds distance (0.093) and Nye similarity score from TreeDist (0.917) further support the robustness of the inferred phylogeny and its genealogical congruence across independent tree-building methods (Supplementary Fig. 3 and online supporting file: IQ-Tree Concordance stats.xlsx in the FigShare repository). This analysis supports the genomic coherence of the taxonomic reassignment and reinforces the reliability of our phylophenetic framework.

Gene predictions using FunGAP identified 13,115 protein-coding genes, 121 tRNA genes, and 69 rRNA genes (Table 1). Of the total coding genes, 5,518 (42.07%) were assigned to a KEGG Orthology (KO) molecular function, a proportion that falls within the expected range of ortholog-annotated genes (30% to 50%) for assembled genomes (Griesemer et al. 2018). Moreover, the identification of KO numbers for some of the proteins encoded in the A3.I.1 genome enabled the reconstruction of 434 pathways. Additionally, 46 molecular functions typical of cytochrome P450 enzymes were detected (see file A3I1_KEGG_annotation.ko in Figshare), which could enable *C. oxysporum* A3.I.1 to metabolize drugs, hormones, and xenobiotics. Notably, 84 complete metabolic modules, related to the metabolisms of carbohydrates, energy, lipids, nucleotides, amino acids, glycans, cofactors and vitamins, and terpenoids and polyketides, were identified (Supplementary Table 2). These modules represent curated sets of enzymatic steps that determine specific biological functions; their reconstruction provides a concise pathway-level view of the functional potential of *C. oxysporum* A3.I.1, reflecting their key biochemical capabilities and enhancing genome annotation beyond individual gene-level assignments.

Notably, a metabolic branch related to the conversion of aromatic nitrogen-containing compound derivatives (thioamides, nitriles, and benzamide) into activated benzoate intermediates was identified, consistent with the annotation of these functions within the KEGG class Aminobenzoate degradation. Within this branch, several sequence features are particularly relevant: a monooxygenase [EC: 1.14.13.] predicted to oxidize thiobenzamide, a xenobiotic-derived compound; a nitrilase (EC: 3.5.5.1) catalyzing the conversion of benzonitrile to benzoate; and an amidase [EC: 3.5.1.4], which transforms benzamide into benzoate. Surprisingly, no nitrile hydratase [EC: 4.2.1.84], the enzyme that transforms benzonitrile to benzamide, was detected. This lack suggests that an equivalent reaction, not yet reported, may account for this reaction (Supplementary Fig. 2) (Holtze et al. 2006; T'Syen et al. 2015; Sulistinah and Sunarko 2020). Interestingly, the nitrilase (EC: 3.5.5.1) and the 4-hydroxybenzoate decarboxylase (EC 4.1.1.61), which transforms phenol to benzoate, are encoded in the strain A3.I.1 genome but not in the control strain AAAS genome (Supplementary Table 2 and Supplementary Fig. 2). These observations may be related to the ability of strain A3.I.1 to degrade PU, a polymer characterized by amide bonds and often containing aromatic structures.

## Supplementary Material

jkag025_Supplementary_Data

## Data Availability

The genome assembly (ASM2101885v1) and sequencing data for *Cladosporium oxysporum* strain A3.I.1 have been submitted to GenBank under the accession number GCA_021018855.1. The Whole-Genome Shotgun (WGS) project is available under the identifier JAIQXY01, as part of BioProject PRJNA661343. Genomic data can be accessed through the National Center for Biotechnology Information databases https://www.ncbi.nlm.nih.gov/. A Data repository including detailed information of the genomic analysis can be found at https://doi.org/10.6084/m9.figshare.29755565.v5 and a list of the different files of this repository is included in Supplementary File 1. The *C. oxysporum* strain A3.I.1 was deposited in the Culture Collection at Cepario Facultad de Química, UNAM, World Data Centre for Microorganisms CFQ100, under the accession number: CFQ-H-246. Supplemental material available at G3 online.
