# Supplementary material for: Chromosome-level assembly and phylophenetic insights of Cladosporium oxysporum A3.I.1, a fungus with the ability to degrade polyurethane polymers
Source: G3 (Bethesda). 2026 Feb 26;16(4):jkag025. doi: 10.1093/g3journal/jkag025 (PMC13042320; doi:10.1093/g3journal/jkag025)
Supplement: jkag025_Supplementary_Data [file jkag025_supplementary_data.zip › File_S1_G3-2025-406424.docx]

A Data repository is available at <https://doi.org/10.6084/m9.figshare.29755565>.

Files available in this repository include:

- A3.I.1_Mitochondrion.gff contains the General Feature Format annotation of the mitochondrial genome for the fungal strain A3.I.1.
- The Quality Assessment A3.I.1 Genome Assembly Report.html offers an HTML report that evaluates the quality of the A3.I.1 genome assembly.
- Uniq Sequences A3.I.1.faa includes unique protein sequences derived from the A3.I.1 genome.
- The Anotation_CDS_FunGap_A3.I.1.faa file lists protein-coding sequences annotated using the FunGap tool, while the corresponding Anotation_CDS_FunGap_A3.I.1.fna contains the nucleotide sequences for these coding regions.
- The C_oxysporum_A3.I.1_Mitogenomev01.fasta file contains the complete mitochondrial genome sequence in FASTA format.
- A brief comparison of hexanucleotide patterns is stored in Hexamer_comparison A3.I1.txt.
- The A3i1q_vs_AAASr.vcf file is the variant call format (VCF) file detailing detected genomic variants in A3.I.1 when compared to a reference strain AAAS_A1.
- Genome-wide annotation results are provided in Fungap_out_A3.I.1.gff3, and a summary of the FunGap annotation process is found in Fungap_out_A3.I.1.html.
- A brief table of telomeric repeat motifs appears in Telomeric repeats_A3.I.1.tsv, and species delimitation partitions based on bPTP are presented in bPTP_heuristic_SupportPartition_A3.I.1.txt.
- A data list of the 41 genomes assemblies used in this study Cladosporium_genomeData.csv.tsv
- Concordance analysis between Figtree and UFCG tree is available in: IQ-Tree Concordance stats.xlsx.
