# Supplementary material for: Chromosome-level assembly and phylophenetic insights of Cladosporium oxysporum A3.I.1, a fungus with the ability to degrade polyurethane polymers
Source: G3 (Bethesda). 2026 Feb 26;16(4):jkag025. doi: 10.1093/g3journal/jkag025 (PMC13042320; doi:10.1093/g3journal/jkag025)
Supplement: jkag025_Supplementary_Data [file jkag025_supplementary_data.zip › Supplementary_Figures_and_Tables_G3-2025-406424.docx]

Supplementary information

**Chromosome-level assembly and phylophenetic insights of *Cladosporium oxysporum* A3.I.1, a fungus with the ability to degrade polyurethane polymers**

**Ayixon Sánchez-Reyes^1*^, Itzayana Chavarría-Quintanilla^2^, Martín Vargas-Suárez^2^, Miguel A. Cevallos^3^, Itzel Gaytán^4^, Herminia Loza-Tavera^2^***

^1^Investigador por México, SECIHTI-Instituto de Biotecnología, Universidad Nacional Autónoma de México, 62210 Cuernavaca, Morelos, México.

^2^Departamento de Bioquímica, Facultad de Química, Universidad Nacional Autónoma de México, 04510 Coyoacán, Ciudad de México, México.

^3^Programa de Genómica Evolutiva, Centro de Ciencias Genómicas, Universidad Nacional Autónoma de México, 62210 Cuernavaca, Morelos, México.

^4^Departamento de Procesos y Tecnología, División de Ciencias Naturales e Ingeniería, Universidad Autónoma Metropolitana, Unidad Cuajimalpa, 05348 Cuajimalpa, Ciudad de México, México.

***Authors for Correspondence:

Herminia Loza-Tavera, hlozat@unam.mx. Departamento de Bioquímica, Facultad de Química, Universidad Nacional Autónoma de México, 04510 Coyoacán, Ciudad de México, México. Phone number: +52 (55) 5622-5280; fax number: +52 (55) 5622-5329

Ayixon Sánchez-Reyes, ayixon.sanchez@ibt.unam.mx. Instituto de Biotecnología, Universidad Nacional Autónoma de México, 62210 Cuernavaca, Morelos, México.


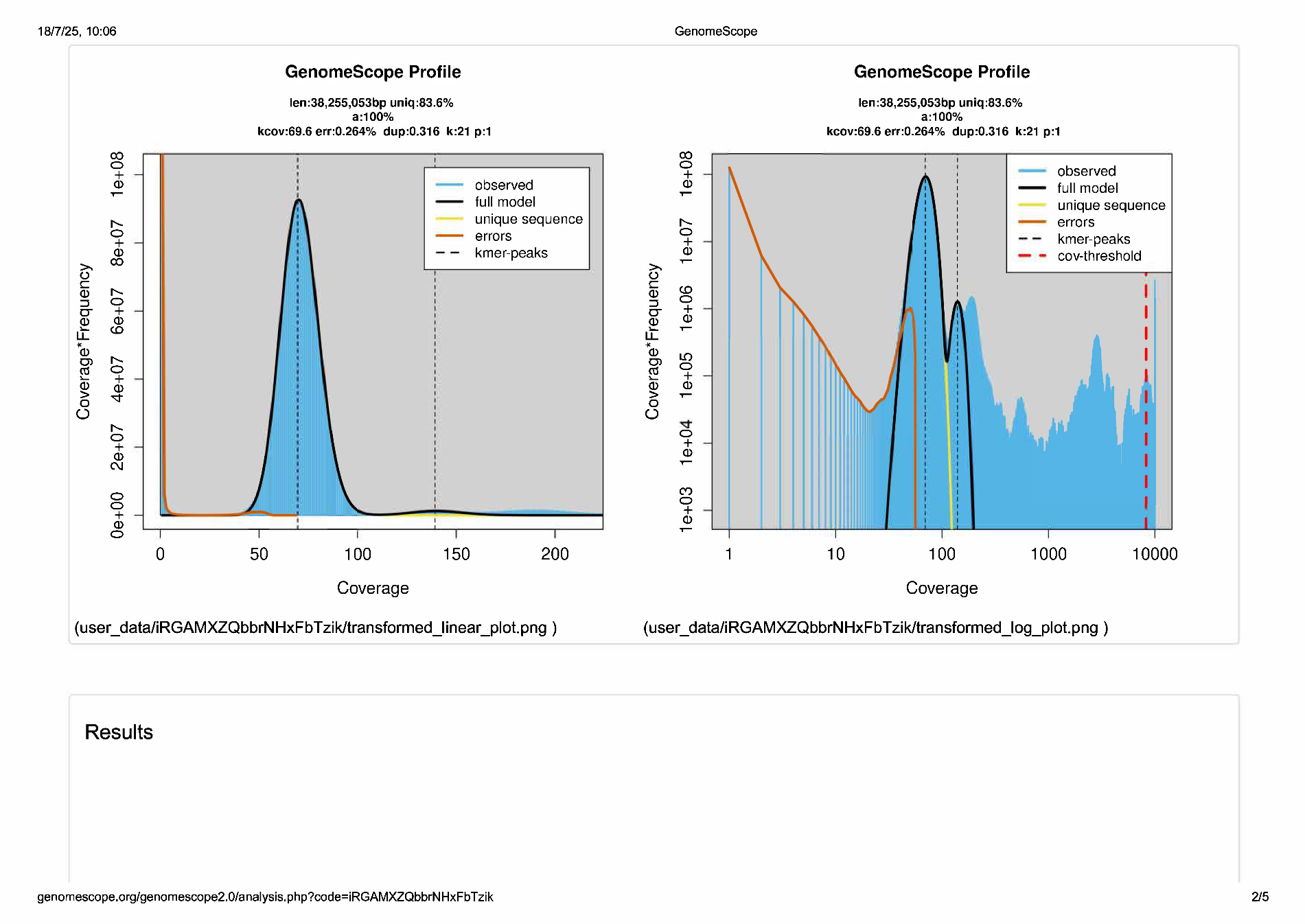


**Figure S1.** K-mer spectrum and fitted models for Cladosporium oxysporum A3.I.1. K-mer plot was generated using Genomescope 2.0 with parameters set as default (k: K-mer length = 21; p:ploidy = 1n).

**Table S1.** GenomeScope 2.0 summary statistics for the analyzed genome.

| Property | Min | Max |
| --- | --- | --- |
| Homozygous (a) | 100% | 100% |
| Estimated Genome Haploid Length | 38,238,204 bp | 38,255,053 bp |
| Estimated Genome Repeat Length | 6,281,029 bp | 6,283,796 bp |
| Estimated Genome Unique Length | 31,957,175 bp | 31,971,257 bp |
| Model Fit | 83.39% | 96.49% |
| Read Error Rate | 0.26% | 0.26% |

**Table S2.** Metabolic potential of *Cladosporium oxysporum* A3.I.1. showed by the complete modules identified by the KEGG Mapper reconstruction analysis. The complete set of Metabolic Modules (84) and Metabolic Pathways (434) identified by the KEGG Mapper reconstruction analysis indicates the presence of all required KO orthologs in the *C. oxysporum* A3.I.1 genome, reflecting the fungus's metabolic potential. Hyperlinks provide direct access to the corresponding KEGG module pages, allowing visualization of the reconstructed metabolic maps and detailed information on the involved reactions or cellular processes.

| Top of Form |
| --- |
| - Pathway (434) |
| - Module (84) |
| Bottom of Form |
| Carbohydrate metabolism |
| Central carbohydrate metabolism |
| - - - [M00001](https://www.genome.jp/dbget-bin/www_bget?md:M00001) Glycolysis (Embden-Meyerhof pathway), glucose => pyruvate |
| - - - [M00002](https://www.genome.jp/dbget-bin/www_bget?md:M00002) Glycolysis, core module involving three-carbon compounds |
| - - - [M00003](https://www.genome.jp/dbget-bin/www_bget?md:M00003) Gluconeogenesis, oxaloacetate => fructose-6P |
| - - - [M00307](https://www.genome.jp/dbget-bin/www_bget?md:M00307) Pyruvate oxidation, pyruvate => acetyl-CoA |
| - - - [M00010](https://www.genome.jp/dbget-bin/www_bget?md:M00010) Citrate cycle, first carbon oxidation, oxaloacetate => 2-oxoglutarate |
| - - - [M00004](https://www.genome.jp/dbget-bin/www_bget?md:M00004) Pentose phosphate pathway (Pentose phosphate cycle) |
| - - - [M00006](https://www.genome.jp/dbget-bin/www_bget?md:M00006) Pentose phosphate pathway, oxidative phase, glucose 6P => ribulose 5P |
| - - - [M00007](https://www.genome.jp/dbget-bin/www_bget?md:M00007) Pentose phosphate pathway, non-oxidative phase, fructose 6P => ribose 5P |
| - - - [M00005](https://www.genome.jp/dbget-bin/www_bget?md:M00005) PRPP biosynthesis, ribose 5P => PRPP |
| Other carbohydrate metabolism |
| - - - [M00081](https://www.genome.jp/dbget-bin/www_bget?md:M00081) Pectin degradation |
| - - - [M00632](https://www.genome.jp/dbget-bin/www_bget?md:M00632) Galactose degradation, Leloir pathway, galactose => alpha-D-glucose-1P |
| - - - [M00854](https://www.genome.jp/dbget-bin/www_bget?md:M00854) Glycogen biosynthesis, glucose-1P => glycogen/starch |
| - - - [M00855](https://www.genome.jp/dbget-bin/www_bget?md:M00855) Glycogen degradation, glycogen => glucose-6P |
| - - - [M00012](https://www.genome.jp/dbget-bin/www_bget?md:M00012) Glyoxylate cycle |
| Energy metabolism |
| Sulfur metabolism |
| - - - [M00176](https://www.genome.jp/dbget-bin/www_bget?md:M00176) Assimilatory sulfate reduction, sulfate => H_2_S |
| ATP synthesis |
| - - - [M00148](https://www.genome.jp/dbget-bin/www_bget?md:M00148) Succinate dehydrogenase (ubiquinone) ( |
| Lipid metabolism |
| Fatty acid metabolism |
| - - - [M00082](https://www.genome.jp/dbget-bin/www_bget?md:M00082) Fatty acid biosynthesis, initiation |
| - - - [M00083](https://www.genome.jp/dbget-bin/www_bget?md:M00083) Fatty acid biosynthesis, elongation |
| - - - [M00415](https://www.genome.jp/dbget-bin/www_bget?md:M00415) Fatty acid elongation in endoplasmic reticulum |
| - - - [M00086](https://www.genome.jp/dbget-bin/www_bget?md:M00086) beta-Oxidation, acyl-CoA synthesis |
| - - - [M00861](https://www.genome.jp/dbget-bin/www_bget?md:M00861) beta-Oxidation, peroxisome, VLCFA |
| Sterol biosynthesis |
| - - - [M00102](https://www.genome.jp/dbget-bin/www_bget?md:M00102) Ergocalciferol biosynthesis, FPP => ergosterol/ergocalciferol |
| Lipid metabolism |
| - - - [M00089](https://www.genome.jp/dbget-bin/www_bget?md:M00089) Triacylglycerol biosynthesis |
| - - - [M00098](https://www.genome.jp/dbget-bin/www_bget?md:M00098) Acylglycerol degradation |
| - - - [M00091](https://www.genome.jp/dbget-bin/www_bget?md:M00091) Phosphatidylcholine (PC) biosynthesis, PE => PC |
| - - - [M00092](https://www.genome.jp/dbget-bin/www_bget?md:M00092) Phosphatidylethanolamine (PE) biosynthesis, ethanolamine => PE ( |
| - - - [M00093](https://www.genome.jp/dbget-bin/www_bget?md:M00093) Phosphatidylethanolamine (PE) biosynthesis, PA => PS => PE |
| - - - [M00094](https://www.genome.jp/dbget-bin/www_bget?md:M00094) Ceramide biosynthesis |
| - - - [M00099](https://www.genome.jp/dbget-bin/www_bget?md:M00099) Sphingosine biosynthesis |
| - - - [M00100](https://www.genome.jp/dbget-bin/www_bget?md:M00100) Sphingosine degradation |
| Nucleotide metabolism |
| Purine metabolism |
| - - - [M00049](https://www.genome.jp/dbget-bin/www_bget?md:M00049) Adenine ribonucleotide biosynthesis, IMP => ADP, ATP |
| - - - [M00050](https://www.genome.jp/dbget-bin/www_bget?md:M00050) Guanine ribonucleotide biosynthesis, IMP => GDP, GTP |
| - - - [M00053](https://www.genome.jp/dbget-bin/www_bget?md:M00053) Deoxyribonucleotide biosynthesis, ADP/GDP/CDP/UDP => dATP/dGTP/dCTP/dUTP |
| - - - [M00958](https://www.genome.jp/dbget-bin/www_bget?md:M00958) Adenine ribonucleotide degradation, AMP => Urate |
| - - - [M00959](https://www.genome.jp/dbget-bin/www_bget?md:M00959) Guanine ribonucleotide degradation, GMP => Urate |
| Pyrimidine metabolism |
| - - - [M00051](https://www.genome.jp/dbget-bin/www_bget?md:M00051) De novo pyrimidine biosynthesis, glutamine (+ PRPP) => UMP |
| - - - [M00052](https://www.genome.jp/dbget-bin/www_bget?md:M00052) Pyrimidine ribonucleotide biosynthesis, UMP => UDP/UTP,CDP/CTP |
| - - - [M00938](https://www.genome.jp/dbget-bin/www_bget?md:M00938) Pyrimidine deoxyribonucleotide biosynthesis, UDP => dTTP ( |
| Amino acid metabolism |
| Serine and threonine metabolism |
| - - - [M00020](https://www.genome.jp/dbget-bin/www_bget?md:M00020) Serine biosynthesis, glycerate-3P => serine |
| - - - [M00018](https://www.genome.jp/dbget-bin/www_bget?md:M00018) Threonine biosynthesis, aspartate => homoserine => threonine ( |
| - - - [M00621](https://www.genome.jp/dbget-bin/www_bget?md:M00621) Glycine cleavage system |
| - - - [M00555](https://www.genome.jp/dbget-bin/www_bget?md:M00555) Betaine biosynthesis, choline => betaine |
| Cysteine and methionine metabolism |
| - - - [M00338](https://www.genome.jp/dbget-bin/www_bget?md:M00338) Cysteine biosynthesis, homocysteine + serine => cysteine |
| - - - [M00017](https://www.genome.jp/dbget-bin/www_bget?md:M00017) Methionine biosynthesis, aspartate => homoserine => methionine |
| - - - [M00034](https://www.genome.jp/dbget-bin/www_bget?md:M00034) Methionine salvage pathway |
| - - - [M00035](https://www.genome.jp/dbget-bin/www_bget?md:M00035) Methionine degradation |
| Branched-chain amino acid metabolism |
| - - - [M00019](https://www.genome.jp/dbget-bin/www_bget?md:M00019) Valine/isoleucine biosynthesis, pyruvate => valine / 2-oxobutanoate => isoleucine |
| - - - [M00570](https://www.genome.jp/dbget-bin/www_bget?md:M00570) Isoleucine biosynthesis, threonine => 2-oxobutanoate => isoleucine |
| - - - [M00432](https://www.genome.jp/dbget-bin/www_bget?md:M00432) Leucine biosynthesis, 2-oxoisovalerate => 2-oxoisocaproate |
| - - - [M00036](https://www.genome.jp/dbget-bin/www_bget?md:M00036) Leucine degradation, leucine => acetoacetate + acetyl-CoA |
| Lysine metabolism |
| - - - [M00030](https://www.genome.jp/dbget-bin/www_bget?md:M00030) Lysine biosynthesis, AAA pathway, 2-oxoglutarate => 2-aminoadipate => lysine |
| - - - [M00433](https://www.genome.jp/dbget-bin/www_bget?md:M00433) Lysine biosynthesis, 2-oxoglutarate => 2-oxoadipate |
| Arginine and proline metabolism |
| - - - [M00028](https://www.genome.jp/dbget-bin/www_bget?md:M00028) Ornithine biosynthesis, glutamate => ornithine |
| - - - [M00844](https://www.genome.jp/dbget-bin/www_bget?md:M00844) Arginine biosynthesis, ornithine => arginine |
| - - - [M00015](https://www.genome.jp/dbget-bin/www_bget?md:M00015) Proline biosynthesis, glutamate => proline |
| - - - [M00970](https://www.genome.jp/dbget-bin/www_bget?md:M00970) Proline degradation, proline => glutamate |
| - - - [M00972](https://www.genome.jp/dbget-bin/www_bget?md:M00972) Proline metabolism |
| Polyamine biosynthesis |
| - - - [M00133](https://www.genome.jp/dbget-bin/www_bget?md:M00133) Polyamine biosynthesis, arginine => agmatine => putrescine => spermidine |
| - - - [M00134](https://www.genome.jp/dbget-bin/www_bget?md:M00134) Polyamine biosynthesis, arginine => ornithine => putrescine ) |
| - - - [M00135](https://www.genome.jp/dbget-bin/www_bget?md:M00135) GABA biosynthesis, eukaryotes, putrescine => GABA |
| Histidine metabolism |
| - - - [M00026](https://www.genome.jp/dbget-bin/www_bget?md:M00026) Histidine biosynthesis, PRPP => histidine |
| Aromatic amino acid metabolism |
| - - - [M00022](https://www.genome.jp/dbget-bin/www_bget?md:M00022) Shikimate pathway, phosphoenolpyruvate + erythrose-4P => chorismate |
| - - - [M00024](https://www.genome.jp/dbget-bin/www_bget?md:M00024) Phenylalanine biosynthesis, chorismate => phenylpyruvate => phenylalanine |
| - - - [M00025](https://www.genome.jp/dbget-bin/www_bget?md:M00025) Tyrosine biosynthesis, chorismate => HPP => tyrosine |
| Other amino acid metabolism |
| - - - [M00027](https://www.genome.jp/dbget-bin/www_bget?md:M00027) GABA (gamma-Aminobutyrate) shunt |
| - - - [M00118](https://www.genome.jp/dbget-bin/www_bget?md:M00118) Glutathione biosynthesis, glutamate => glutathione |
| Glycan metabolism |
| Nucleotide sugar biosynthesis |
| - - - [M00892](https://www.genome.jp/dbget-bin/www_bget?md:M00892) UDP-GlcNAc biosynthesis, eukaryotes, Fru-6P => UDP-GlcNAc |
| - - - [M00549](https://www.genome.jp/dbget-bin/www_bget?md:M00549) UDP-Glc biosynthesis, Glc => UDP-Glc ( |
| - - - [M00554](https://www.genome.jp/dbget-bin/www_bget?md:M00554) UDP-Gal biosynthesis, Gal => UDP-Gal |
| - - - [M01000](https://www.genome.jp/dbget-bin/www_bget?md:M01000) GDP-Man biosynthesis, Fru-6P => GDP-Man |
| Glycan biosynthesis |
| - - - [M00055](https://www.genome.jp/dbget-bin/www_bget?md:M00055) N-glycan precursor biosynthesis |
| - - - [M00072](https://www.genome.jp/dbget-bin/www_bget?md:M00072) N-glycosylation by oligosaccharyltransferase |
| - - - [M00073](https://www.genome.jp/dbget-bin/www_bget?md:M00073) N-glycan precursor trimming |
| Metabolism of cofactors and vitamins |
| Cofactor and vitamin metabolism |
| - - - [M00916](https://www.genome.jp/dbget-bin/www_bget?md:M00916) Pyridoxal-P biosynthesis, R5P + glyceraldehyde-3P + glutamine => pyridoxal-P |
| - - - [M00912](https://www.genome.jp/dbget-bin/www_bget?md:M00912) NAD biosynthesis, tryptophan => quinolinate => NAD |
| - - - [M00120](https://www.genome.jp/dbget-bin/www_bget?md:M00120) Coenzyme A biosynthesis, pantothenate => CoA |
| - - - [M00123](https://www.genome.jp/dbget-bin/www_bget?md:M00123) Biotin biosynthesis, pimeloyl-ACP/CoA => biotin |
| - - - [M00881](https://www.genome.jp/dbget-bin/www_bget?md:M00881) Lipoic acid biosynthesis, plants and bacteria, octanoyl-ACP => dihydrolipoyl-E2/H |
| - - - [M00880](https://www.genome.jp/dbget-bin/www_bget?md:M00880) Molybdenum cofactor biosynthesis, GTP => molybdenum cofactor |
| - - - [M00141](https://www.genome.jp/dbget-bin/www_bget?md:M00141) C1-unit interconversion, eukaryotes |
| - - - [M00868](https://www.genome.jp/dbget-bin/www_bget?md:M00868) Heme biosynthesis, animals and fungi, glycine => heme |
| - - - [M00128](https://www.genome.jp/dbget-bin/www_bget?md:M00128) Ubiquinone biosynthesis, eukaryotes, 4-hydroxybenzoate + polyprenyl-PP => ubiquinone |
| Biosynthesis of terpenoids and polyketides |
| Terpenoid backbone biosynthesis |
| - - - [M00095](https://www.genome.jp/dbget-bin/www_bget?md:M00095) C5 isoprenoid biosynthesis, mevalonate pathway |
| - - - [M00367](https://www.genome.jp/dbget-bin/www_bget?md:M00367) C10-C20 isoprenoid biosynthesis, non-plant eukaryotes |


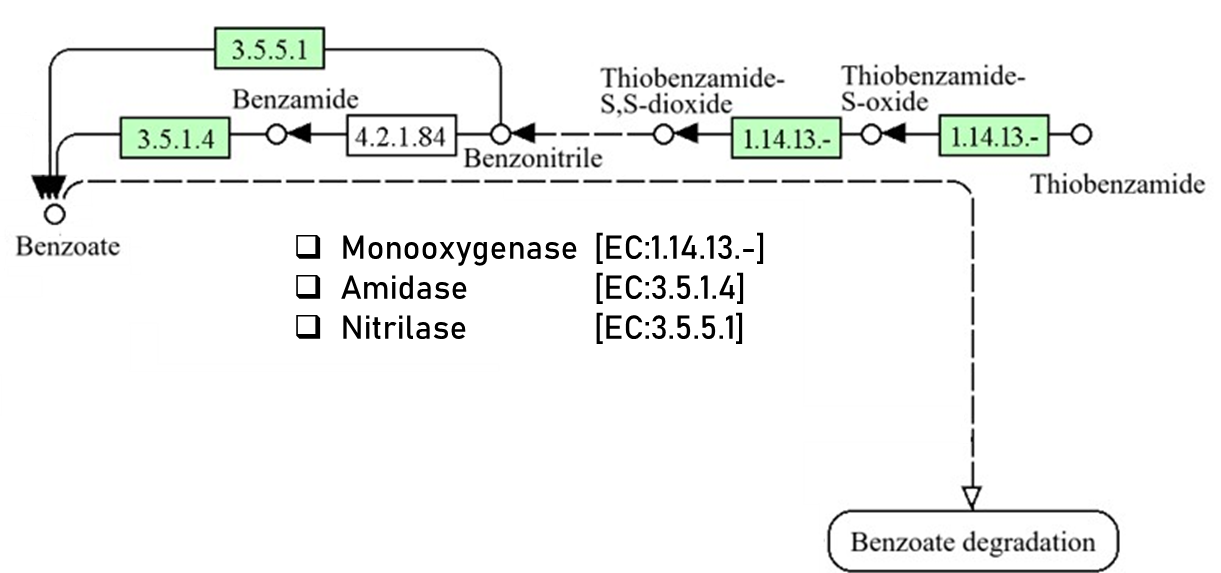


**Figure S2.** A complete pathway for the biodegradation of thiobenzamide to benzoate in the aminobenzoate degradation route was detected encoded in the Cladosporium oxysporum A3.I.1 genome, suggesting the potential for the utilization of PU degradation products as carbon sources by conversion into activated benzoate intermediates. Numbers in boxes correspond to E.C. numbers. Green and white boxes indicate enzymes encoded and not encoded, respectively, in the A3.I.1 genome.


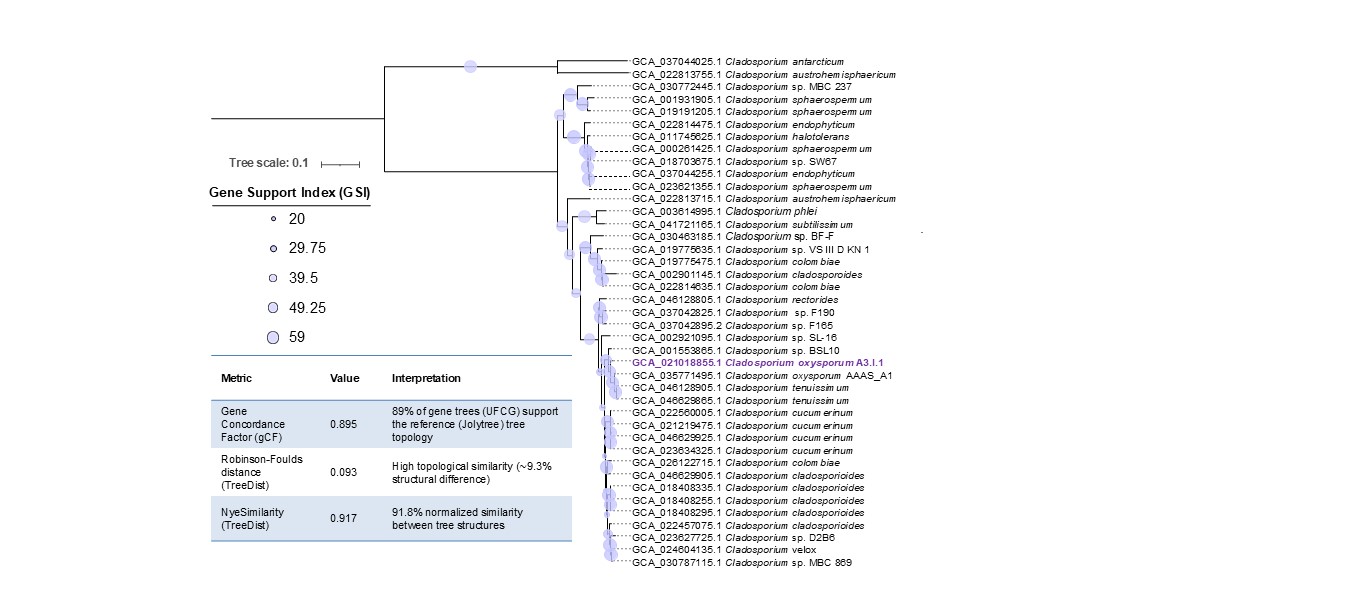


**Figure S3.** UFCG tree reconstructed from 59 conserved genes across 41 *Cladosporium* genomes. Branch support is measured by the Gene Support Index (GSI), which counts the number of individual gene trees supporting each bipartition. Phylogenetic concordance metrics are also presented, comparing this tree to one inferred using JolyTree, which relies on transformed genomic distances.
